# Supplementary material for: The causal relationship between the human gut microbiota and pyogenic arthritis: a Mendelian randomization study
Source: Front Cell Infect Microbiol. 2024 Nov 26;14:1452480. doi: 10.3389/fcimb.2024.1452480 (PMC11629706; doi:10.3389/fcimb.2024.1452480)
Supplement: Supplementary file 1 [file DataSheet1.zip › mendelian test/ebi-a-GCST90027543.csv_forest.pdf]

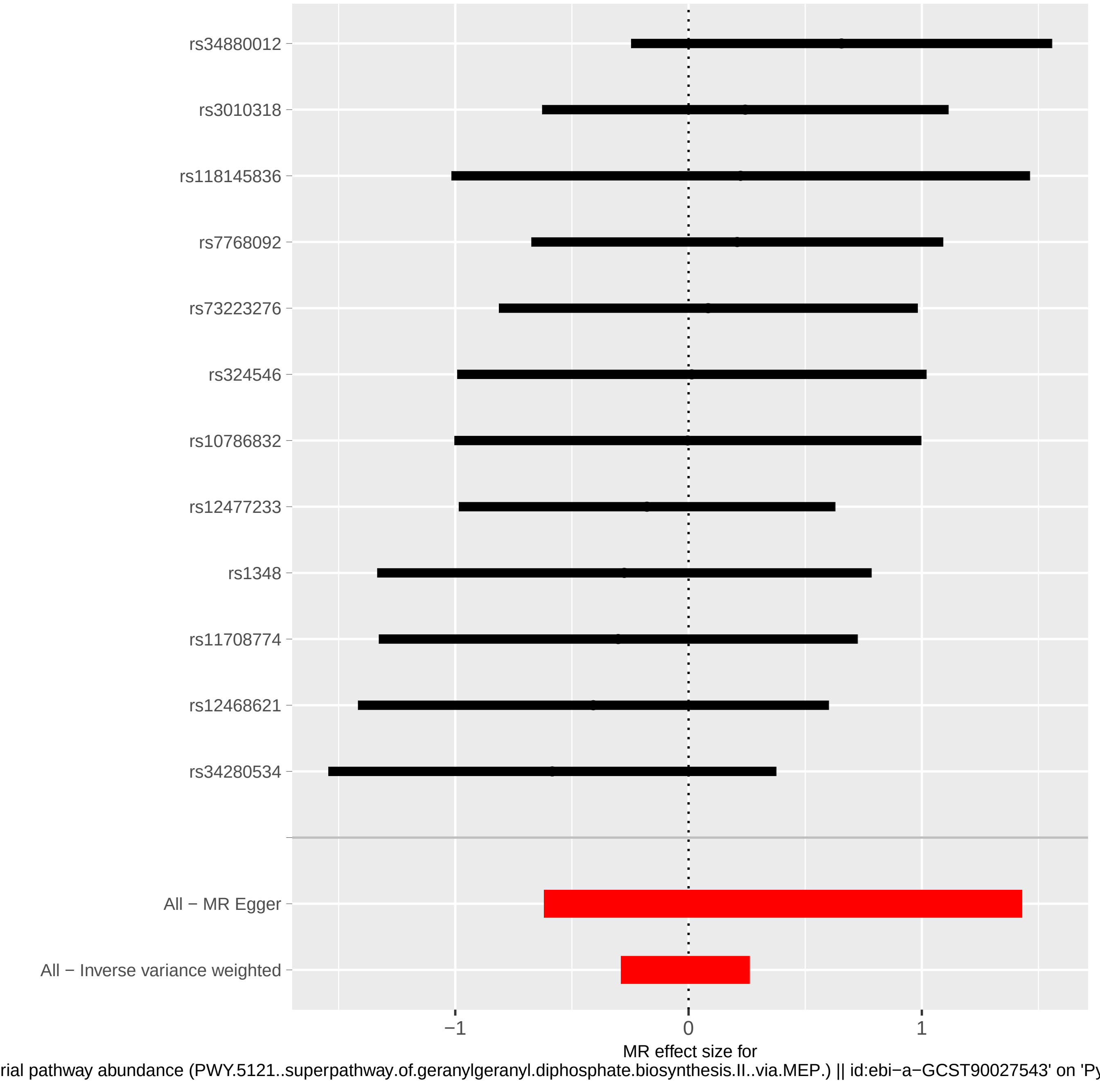

rs34880012
rs3010318
rs118145836
rs7768092
rs73223276
rs324546
rs10786832
rs12477233
rs1348
rs11708774
rs12468621
rs34280534
All − MR Egger
All − Inverse variance weighted
−1
0
1
MR effect size for
rial pathway abundance (PWY.5121..superpathway.of.geranylgeranyl.diphosphate.biosynthesis.II..via.MEP.) || id:ebi−a−GCST90027543' on 'Py
